# Draft Genome Sequence of Streptomyces moderatus DT446, Isolated from Root Nodules of Casuarina cunninghamiana

**DOI:** 10.1128/mra.00181-23

**Published:** 2023-07-11

**Authors:** Thirumagal Thirugnanam, Dhanasekaran Dharumadurai, Olubukola Oluranti Babalola

**Affiliations:** a Department of Microbiology, Bharathidasan University, Tiruchirappalli, Tamil Nadu, India; b Food Security and Safety Focus Area, Faculty of Natural and Agricultural Sciences, North-West University, Mmabatho, South Africa; The University of Arizona

## Abstract

A putative plant growth-promoting endophytic Streptomyces moderatus strain, DT446, was isolated from the root nodules of Casuarina cunninghamiana in Tamil Nadu, India. We report a draft genome sequence for S. moderatus DT446, with 8,168,245 bp and a GC content of 70.9%.

## ANNOUNCEMENT

Actinobacteria represent 20 to 30% of rhizospheric communities and include Gram-positive, high-GC-content (>50%), soil-dwelling organisms that play a significant role in nitrogen fixation (1). *Frankia* strains are endosymbionts in dicotyledonous plants that can enhance plant growth, solubilize phosphorus, and liberate indole-3-acetic acid (IAA) and siderophores (2–5). Non-*Frankia* actinobacterial species, including *Micromonospora*, *Streptomyces*, and *Actinoplanes* species, can also act as helper bacteria that stimulate plant growth. This genome study aims to improve our knowledge of the flexibility of non-*Frankia* actinobacteria and their adaptive roles in plant growth promotion (PGP) and symbiosis (6). Streptomyces moderatus DT446 was isolated from the root nodules of the actinorhizal plant Casuarina cunninghamiana in Tamil Nadu, India (sampling location coordinates: 11°26′16.476″N, 79°43′9.84″E), sampled on 1 September 2021. Surface-sterilized root nodules were homogenized and plated on starch casein agar (SCA) (7). DNA was extracted from a purified culture that had been grown on SCA at 27°C for 7 days using an Xploregen kit (Bengaluru, Karnataka, India), and quantification was performed with a Qubit fluorometer. A double-stranded DNA (dsDNA) high-sensitivity (HS) assay kit was used to quantify DNA with a Qubit 3 fluorometer. One hundred nanograms of DNA was sheared into 200- to 300-bp fragments using the KAPA fragmentation process. Using a HyperPlus end repair and A-tailing enzyme mixture, the fragmented samples were treated for end repair and A-tailing. Adapters were added and ligated to the end-repaired DNA fragments using DNA ligase, immediately after the end repair and A-tailing, and fragments were purified using AMPure beads. A sequencing library was prepared using the NEBNext Ultra II DNA library preparation kit and amplified using the NEBNext Ultra II Q5 Master Mix, following the manufacturer’s protocol. The resulting DNA library was eluted in 15 μL of 0.1× Tris-EDTA (TE) buffer and quantified using a dsDNA HS kit. Fragment analysis was carried out with an Agilent DNA 7500 chip, and an Illumina HiSeq 4000 system was used for sequencing, which generated 56.6 million reads and 9,913,140 bp of total sequenced data. FastQC and MultiQC were used for quality control (8, 9). Quality trimming was applied to the raw reads using Trim Galore v0.6.4 (10). The quality-trimmed data were then used for the assembly; the tool used for the bacterial assembly was Unicycler v0.4.8 (11). Using the assembled file and the multilocus sequence typing (MLST) method (with PubMLST), the organism was confirmed and the related reference sequence was downloaded from NCBI. After the initial assembly, fasta files obtained with *n* numbers of contigs were further aligned with the reference sequence (https://www.ncbi.nlm.nih.gov/taxonomy/1851167) using CONTIGuator (12). Streptomyces moderatus was identified by sequence analysis as having 23 contigs, an 8,168,245-bp genome, and *N*_50_ and *L*_50_ values of 546,797 bp and 5, respectively. Numerous strains of the genus *Streptomyces* have been reported to stimulate plant growth in the phylum *Actinobacteria* (7, 13). The NCBI Prokaryotic Genome Annotation Pipeline (PGAP) v5.3 predicted 7,206 protein-coding genes, 1 rRNA gene, 71 tRNA genes, 3 noncoding RNA genes, and 109 pseudogenes (14). Computational analysis of the genome of S. moderatus using PGAP revealed the presence of *nod* and *hup* genes, as well as genes involved in nitrogen metabolism, IAA, cytokinin, siderophore, and hydrogen cyanide production, osmotic stress and cold and heat shock responses, and phosphate solubilization, as illustrated in Table 1. All of the bioinformatic tools were run with default parameters unless otherwise specified. The genome properties of S. moderatus have experimental confirmation of PGP activities under laboratory conditions. Therefore, the genes reported above may play substantial roles in the PGP of Casuarina cunninghamiana.

**TABLE 1:** Distribution of symbiosis and PGP genes in the genome of S. moderatus

Pathway and gene^*a*^	Product
Nodulation	
*nodD2*	Nodulation protein D2
*nrgA_1*	Ammonium transporter
Hydrogen uptake	
*hup*	DNA-binding protein HU
Phosphate solubilization	
*phoU2*	Phosphate-specific transport system accessory protein PhoU
*ppx1_1*	Exopolyphosphatase 1
Phosphate transport	
*pstP*	PP2C family Ser/Thr phosphatase
*pstA1*	Phosphate transport system permease protein PstA1
*pstC2*	Phosphate transport system permease protein PstC2
*pstS3*	Phosphate-binding protein PstS3
*ugpA*	*sn*-Glycerol-3-phosphate transport system permease protein UgpA
*pstB1*	Phosphate import ATP-binding protein PstB
IAA production	
*trpA*	Tryptophan synthase α-chain
*trpB*	Tryptophan synthase β-chain
*trpC*	Indole-3-glycerol phosphate synthase
*trpE*	Anthranilate synthase component 1
*trpS_1*	Tryptophan-tRNA ligase
*trpG*	Anthranilate synthase component 2
*trpD_2*	Anthranilate phosphoribosyl transferase
Dissimilatory nitrate reduction	
*nasD*	Nitrite reductase [NAD(P)H]
*nirD*	Nitrite reductase (NADH) small subunit
*ntaA_1*	Nitrilotriacetate monooxygenase component A
*norW*	Nitric oxide reductase FlRd-NAD^+^ reductase
*inhA_2*	Isonitrile hydratase
*ntaA_2*	Nitrilotriacetate monooxygenase component A
*nasC*	Assimilatory nitrate reductase catalytic subunit
*nasB*	Assimilatory nitrate reductase electron transfer subunit
*napA_1*	Periplasmic nitrate reductase
*nrtD*	Nitrate import ATP-binding protein NrtD
*narX_1*	Nitrate reductase-like protein NarX
*narY*	Respiratory nitrate reductase 2 β-chain
*narK*	Nitrate/nitrite transporter NarK
*narL*	Putative transcriptional regulatory protein
*narG_1*	Nitrate reductase α-subunit
*narG_2*	Nitrate reductase α-subunit
*narG_3*	Nitrate reductase α-subunit
*nadE*	Glutamine-dependent NAD^+^ synthetase
Cytokinin production	
*miaB*	tRNA-2-methylthio-*N*^6^-dimethylallyladenosine synthase
*miaA*	tRNA dimethylallyltransferase
Sulfur metabolism	
*sufT*	Fe-S protein maturation auxiliary factor SufT
*sufC*	Vegetative protein 296
*sufS_1*	Cysteine desulfurase SufS
ACC deaminase activity	
*accD3*	Putative acetyl-coenzyme A carboxylase carboxyl transferase subunit β
*accA1_1*	Acetyl-/propionyl-coenzyme A carboxylase α-chain
*accD5*	Putative propionyl-CoA carboxylase β-chain 5
Chitinolytic activity	
*chiD*	Chitinase D
*chtA_1*	Chitinase 63
*chiA2*	Chitinase
Nitrogen metabolism	
*glnQ_1*	Glutamine transport ATP-binding protein GlnQ
*glnA_1*	Glutamine synthetase
*glnB_1*	Glutamine synthetase
Ammonia assimilation	
*gltD_1*	Glutamate synthase (NADPH) small chain
*gltB*	Glutamate synthase (NADPH) large chain
*gltA2*	Citrate synthase 1
*gltC_1*	HTH-type transcriptional regulator GltC
*gltX*	Glutamate-tRNA ligase
*gltR*	HTH-type transcriptional regulator GltR
Siderophore production	
*irtA*	Iron import ATP-binding/permease protein IrtA
*fadF*	Putative iron-sulfur-binding oxidoreductase FadF
*ideR*	Iron-dependent repressor IdeR
*fieF*	Ferrous-iron efflux pump FieF
*yfiZ*	Putative siderophore transport system permease protein YfiZ
*yfhA*	Putative siderophore transport system permease protein YfhA
Stress regulators	
*aqpZ*	Aquaporin Z osmotic
*cspA*	Cold shock protein CspA
*hspA*	Spore protein SP21
*hspR*	Putative heat shock protein HspR
*hspX*	α-Crystallin
Nitrogenase enzyme protection	
*hpnE*	Hydroxysqualene dehydroxylase
*shc*	Squalene-hopene cyclase

a ACC, 1-aminocyclopropane-1-carboxylate; HTH, helix-turn-helix.

### Data availability.

This draft genome project and associated data have been deposited in the NCBI database under the accession number CP116671, with BioProject accession number PRJNA925685, BioSample accession number SAMN32805376, and SRA accession number SRR23145503.
